# Short-Term Effects of Climate Change on Planktonic Heterotrophic Prokaryotes in a Temperate Coastal Lagoon: Temperature Is Good, Ultraviolet Radiation Is Bad, and CO_2_ Is Neutral

**DOI:** 10.3390/microorganisms11102559

**Published:** 2023-10-14

**Authors:** Ana B. Barbosa, Benjamin A. Mosley, Helena M. Galvão, Rita B. Domingues

**Affiliations:** CIMA—Centre for Marine and Environmental Research & ARNET—Infrastructure Network in Aquatic Research, Campus de Gambelas, University of Algarve, 8005-139 Faro, Portugal

**Keywords:** acidification, ultraviolet radiation, warming, heterotrophic prokaryotes, growth, mortality

## Abstract

Planktonic heterotrophic prokaryotes (HProks) are a pivotal functional group in marine ecosystems and are highly sensitive to environmental variability and climate change. This study aimed to investigate the short-term effects of increasing carbon dioxide (CO_2_), ultraviolet radiation (UVR), and temperature on natural assemblages of HProks in the Ria Formosa coastal lagoon during winter. Two multi-stressor microcosm experiments were used to evaluate the isolated and combined effects of these environmental changes on HProk abundance, production, growth, and mortality rates. The isolated and combined effects of increased CO_2_ on HProks were not significant. However, HProk production, cellular activity, instantaneous growth rate, and mortality rate were negatively influenced by elevated UVR and positively influenced by warming. Stronger effects were detected on HProk mortality in relation to specific growth rate, leading to higher HProk net growth rates and abundance under elevated UVR and lower values under warming conditions.

## 1. Introduction

Following an anthropogenically derived increase in the rate of climate change in the 20th century, concerns are mounting regarding the impact that this will have on marine ecosystems, at both local and global levels. Anthropogenically enhanced climate change has contributed to sea surface warming, intensified upper ocean stratification, increased CO_2_ levels, and consequential ocean acidification [1,2]. Other climate change impacts include alterations in irradiance from changing cloud cover or reducing ice sheets and higher ultraviolet irradiance from ozone depletion brought on by the increase in greenhouse gases [3,4].

Heterotrophic prokaryotes (HProks) are key components of marine pelagic food webs, being responsible for the transformation of non-living dissolved and particulate organic matter into living biomass and representing a large fraction of secondary production and a trophic link to metazoans [5]. They rapidly respond to environmental changes, and the impact of climate change on marine ecosystems will heavily depend on the responses of HProks and other marine microbes [6]. However, the prediction of climate change impacts on HProks is a challenging task, since climate-related environmental changes act directly and indirectly on HProks, affecting both bottom-up controls (i.e., growth regulation) and top-down controls (i.e., mortality regulation), sometimes with apparently contrasting outcomes [7]. Furthermore, multiple lines of evidence show that concurrent environmental changes may have interactive, synergistic, or antagonistic effects on marine microbes, thus generating variable responses [8,9].

Temperature is a key determinant of metabolic rates, and experimental warming has been associated with the direct stimulation of HProk growth rates and production [2]. In fact, shifts towards smaller [10], more heterotrophic, bacteria-based food webs [11,12] have been reported under warming scenarios. However, the influence of temperature on HProks is context-dependent, being modulated by other environmental conditions, including substrate availability [13,14], and indirectly affected by the warming impacts on nutrient availability, phytoplankton activity [7] and HProk mortality. In fact, the direct stimulation of predator activity or the influence of trophic cascades has been used to support detrimental effects of experimental warming on HProks [7,15,16] and supports the need to use natural communities to evaluate the impacts of climate change [12,17].

In contrast with warming, the effects of increased CO_2_ on HProks are usually considered mostly indirect and related to the stimulation of phytoplankton activity and subsequent exudation of dissolved organic matter [18]. This apparent resiliency can also be explained by more efficient pH regulatory mechanisms [2]. However, data on the responses of HProks to experimental acidification are not consistent; sometimes, contrasting responses have also been reported in micro- and mesocosm experiments. These variable outcomes, a result of the integrated responses of HProks, phytoplankton, phagotrophic protists, and viruses, constrain the discrimination of direct and indirect effects of acidification on HProks, limiting their prediction [11,18,19,20].

Heterotrophic prokaryotes are amongst the most sensitive organisms to UVR stress due to their high surface to volume ratio, low internal self-shading due to small cell sizes, and lack of photoprotective pigments, which are widespread among phytoplankton [21,22]. However, as for warming and acidification, experimental results have reported contrasting effects of elevated UVR on HProks, including direct inhibition due to HProk cell damage or indirect reductions in phytoplankton production, minor effects, and stimulation due to indirect photochemical processes improving DOM lability [23,24] or UVR-mediated reductions in HProk mortality [25,26].

HProk responses to climate change are therefore complex, context-dependent, shaped by natural environmental conditions and communities, and very difficult to generalize to specific ecosystems such as the Ria Formosa, a shallow, multi-inlet, coastal lagoon system situated on the south coast of Portugal. This productive ecosystem is responsible for about 90% of the Portuguese bivalve production, showing high ecological and socio-economic relevance [27]. However, the Ria Formosa is subjected to various anthropogenic stressors [28] and is located in an area particularly susceptible to climate change [29]. Previous studies addressing climate change impacts on planktonic microbes in the Ria Formosa lagoon have evaluated the short-term effects of isolated and combined changes in sea surface temperature, CO_2_, and UVR on phytoplankton and microzooplankton grazers [30,31,32]. However, no information is currently available on the influence of climate change on heterotrophic prokaryotes, a critical functional group in this lagoon system [27,33]. In this context, the current study aimed to evaluate the short-term effects of increasing temperature, CO_2_, and UVR on natural assemblages of heterotrophic prokaryotes in the Ria Formosa coastal lagoon during the colder, winter period. Two multi-stressor microcosm experiments were used for evaluating the isolated and combined effects of the aforementioned environmental changes on HProk abundance, production, cell-specific activity, specific and net growth rates, and mortality. The working hypothesis, therefore, is that HProk assemblages will benefit from an increase in seawater temperature and UVR, with some potential counteracting effects of CO_2_ increase. Overall, it is expected that changing these environmental stressors will benefit the HProk assemblages.

## 2. Materials and Methods

### 2.1. Study Site

**Figure 1:**
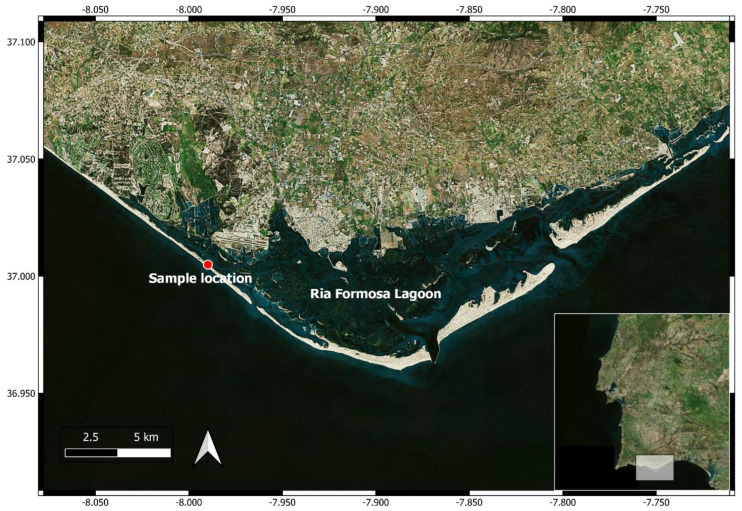
Location of Ria Formosa coastal lagoon system and sampling site (red circle) (made using QGIS 3.18).

The Ria Formosa lagoon is a shallow (mean depth = 2 m), euryhaline, multi-inlet coastal lagoon system located on the southern coast of Portugal, separated from the Atlantic Ocean by sandy barrier islands and peninsulas (Figure 1). Tides are mesotidal and semidiurnal, and the water column is well mixed, with no significant haline or thermal stratification. The adjacent coastal region is affected by regular upwelling events whose effects may extend up to 6 km upstream through lagoon inlets [34]. This system is subjected to a Mediterranean climate, with hot, dry summers, and mild winters, and located in a region extremely vulnerable to climate change [29].

### 2.2. Sampling and Experimental Setup

Two experiments were carried out using water samples collected in the winter (January–February 2012) at an inner location of the Ria Formosa lagoon. For both experiments, sub-surface water samples were collected using 10 L polycarbonate bottles, at low tide, to minimize the influence of the adjacent coastal waters. Water temperature was measured in situ using a YSI 30 probe (Yellow Spring Instruments, Yellow Springs, OH, USA). Water samples used in the experiments were not pre-screened to remove grazers, given that this procedure also removes phytoplankton and phagotrophic protists, increasing the problems associated with the extrapolation of results to the natural ecosystem [35].

Experiment 1 was conducted on 23–25 January 2012 and tested the effects of increases in CO_2_ and UVR on natural assemblages of HProks. Water samples were transferred to translucent 4.5 L UVR-transparent LDPE cubitainers (Nalgene I-Chem Certified Series; Thermo Fisher Scientific, Waltham, MA, USA), with a diffusive CO_2_ loss of 2.9 Pa d^–1^ [36]. A multifactorial experimental regime was prepared, with two spectral treatments (Photosynthetic Available Radiation [PAR] and PAR+UVR) and two CO_2_ treatments (ambient CO_2_ and high CO_2_). For the spectral treatment PAR+UVR, cubitainers were covered with a fishing net that allowed the transmission of 86% of incident solar radiation. For the spectral treatment PAR, cubitainers were covered with a UV-absorbing film (Llumar SHE ER PS7) that allowed the transmission of 87% of incident PAR and eliminated >99% of UVR. PAR intensity was measured with a LI-COR LI-193 spherical underwater quantum sensor. The high CO_2_ treatment was prepared by adding CO_3_^2–^ (as Na_2_CO_3_), HCO_3_^–^ (as NaHCO_3_), and HCl 0.01 N to increase CO_2_ partial pressure (pCO_2_) to the levels expected for the year 2100, according to [37]. pCO_2_ was estimated using the Seacarb package for R (https://www.r-project.org/, accessed 5 June 2023) [38]. Alkalinity, determined by titration [39], and pH, measured with a pH meter (VWR pHenomenal pH1000L, Avantor, Radnor, PA, USA), were used as input variables. At the beginning of incubation, pCO_2_ and pH values were 435.1 µatm and 8.1 in the ambient CO_2_ treatments; and they were 712.1 µatm and 7.9 in the high CO_2_ treatments. At the end of incubation, values for the ambient CO_2_ treatments were 384.0 ± 21.0 µatm and 8.1 ± 0.0; and they were 648.1 ± 21.0 µatm and 7.9 ± 0.0 for the high CO_2_ treatments. 

Four different experimental treatments, prepared in triplicate and incubated outdoors for 48 h inside a tank filled with water, were thus obtained: control (ambient CO_2_, PAR only), CO_2_ (high CO_2_, PAR only), UVR (ambient CO_2_, PAR+UVR), and CO_2_+UVR (high CO_2_, PAR+UVR). At the beginning and end of incubation, water samples were collected for all treatments and replicates for the determination of pH, alkalinity, and abundance of HProks. The net production of HProks was also determined at the end of the experiment.

Experiment 2 was carried out on 6–8 February 2012 and tested the effects of acidification and warming on natural assemblages of HProks. A multifactorial regime was prepared, with two CO_2_ treatments (ambient CO_2_ and high CO_2_) and two temperature (T) treatments (in situ T and high T). The high CO_2_ treatments were prepared as described above. At the beginning of incubation, pCO_2_ and pH values were 389.7 ± 77.0 µatm and 8.1 ± 0.1 in the ambient CO_2_ treatments; and they were 712.3 ± 82.8 µatm and 7.9 ± 0.0 in the high CO_2_ treatments. At the end of incubation, values for the ambient CO_2_ treatments were 379.4 ± 15.5 µatm and 8.1 ± 0.0; and they were 719.0 ± 59.2 µatm and 7.9 ± 0.0 for the high CO2 treatments. Each CO_2_ treatment was incubated under two different temperatures, using a plant growth chamber (Fitoclima S600, Aralab, Rio de Mouro, Portugal) with controlled temperature: ambient T at 10 °C and high T at 13 °C, based on predictions for the southern coast of Portugal to be reached by 2080–2100 [40].

Four different experimental treatments, prepared in triplicate in 2 L polycarbonate bottles (Nalgene), were thus obtained: control (ambient CO_2_, in situ T), CO_2_ (high CO_2_, in situ T), T (ambient CO_2_, high T), and CO_2_+T (high CO_2_, high T). Aliquots were collected at the beginning and end (after 48 h) of incubation for the determination of pH, alkalinity, and abundance of HProks. The net production of HProks was also determined at the end of the experiment.

No nutrients were added to any of the experiments, as we did not expect nutrient limitation in the Ria Formosa coastal lagoon during winter [41]. In both experiments, experimental units (cubitainers or polycarbonate bottles) were manually homogenized twice per day to prevent settlement of cells. All material used in the sampling and experimental procedures was previously washed with HCl 10% and thoroughly rinsed with deionized water.

### 2.3. Analytical Methods

The abundance of HProks was determined using epifluorescence microscopy, following [42]. Samples were preserved with particle-free glutaraldehyde 25% (final concentration 0.2%) immediately after collection and refrigerated until processed. Within 48 h of sampling, samples were filtered (<100 mm Hg) onto 0.2 µm of black polycarbonate membrane filters, mounted on 0.45 µm cellulose acetate backing filters, and stained with acridine orange. Slides were prepared using glass slides and non-fluorescent immersion oil (Cargille Type A); then, they were frozen (−20 °C) until analysis. Observation was made with a Leica DM LB epifluorescence microscope (Wetzlar, Germany), equipped with blue and green light, at 1250× magnification. A minimum of 50 random visual fields, and at least 400 cells, were enumerated for each slide. The biovolume of HProk cells was estimated according to [43], and cell volume (CV, µm^3^ cell^−1^) was used to estimate cell carbon content (CC, fgC cell^−1^) as,

$$CC=0.12\times {CV}^{0.72}$$

using the allometric relationship of [44]. The abundance and mean cell carbon content were used to estimate the biomass of HProks (HProkB).

HProk (carbon) production (HProkP) was determined using the incorporation of ^14^C-leucine [45]. Three sample aliquots, plus three formaldehyde-killed blanks (2% final concentration), were inoculated with ^14^C-leucine (specific activity 304 mCi mmol^–1^, Amersham, 60 nM final concentration), and incubated in the dark, for 2 h, at in situ temperature. The incubation was stopped with the addition of formaldehyde (2% final concentration), and samples and blanks were filtered onto 0.2 μm cellulose nitrate membrane filters and rinsed five times with 5% ice-cold trichloracetic acid (TCA). Dry filters were transferred to scintillation vials with scintillation cocktail (UniverSol^TM^, MP Biomedicals, Santa Ana, CA, USA), and radioactivity was measured on a liquid scintillation counter (Beckman). The disintegrations per minute (DPM) of blanks was subtracted from the mean DPM of the respective samples, and the resultant was used to estimate leucine incorporation rates into the TCA-insoluble fraction. Leucine incorporation rates normalized to HProk abundance were used as an index of cell-specific activity [46]. Since no empirical leucine-to-carbon conversion factors are available for the study area, a theoretical value of 1.5 kg C mol leucine^−1^ was used for converting leucine uptake rates into HProk carbon production [47].

### 2.4. Data Analysis

Specific instantaneous growth rates of HProk assemblages (SGR, d^–1^) were estimated using HProk biomass (µg C L^–1^) and net production (µg C L^−1^ d^−1^) data, assuming exponential growth, according to [46]:

$$SGR=ln\left(1+\left(\frac{HProkP}{HprokB}\right)\right)$$

Net growth rates (NGR, d^−1^) of Hproks were calculated assuming exponential growth, as

$$NGR=\frac{\ln N_{t}-\ln N_{0}}{t}$$

where *N_t_* and *N*_0_ represent the abundance at the end and at the beginning of the experiment, and *t* is the incubation time (2 days for both experiments). Assemblage growth rates should be considered conservative due to the presence of dead or metabolically inactive cells (e.g., [48]). Mortality rates (MR, d^−1^) of Hprok assemblage were indirectly estimated, for each experimental treatment and replicate, as the difference between the SGR and the NGR [49].

Statistical analysis was carried out by first assessing data normality and homogeneity of variances, tested with Shapiro–Wilk and Levene’s tests, respectively. As the assumptions were met, two-way analysis of variance (two-way ANOVA) was used to assess the main effects of the independent variables (CO_2_ and UVR for experiment 1, and CO_2_ and T for experiment 2) and interaction effects (CO_2_ × UVR and CO_2_ × T) upon Hprok abundance, biomass, production, cellular activity, NGR, SGR, and MR. Effect sizes were assessed using omega-squared statistics (ω_G_^2^), which indicates the percentage of variation in the dependent variable attributable to the independent variable [50,51]. Generalized partial ω_G_^2^ values > 0.70 were considered indicative of large effects sizes. All statistical analyses were performed with IBM SPSS^®^ Statistics v. 28 software (Armonk, NY, USA), considering a 0.05 significance level.

## 3. Results

**Figure 2:**
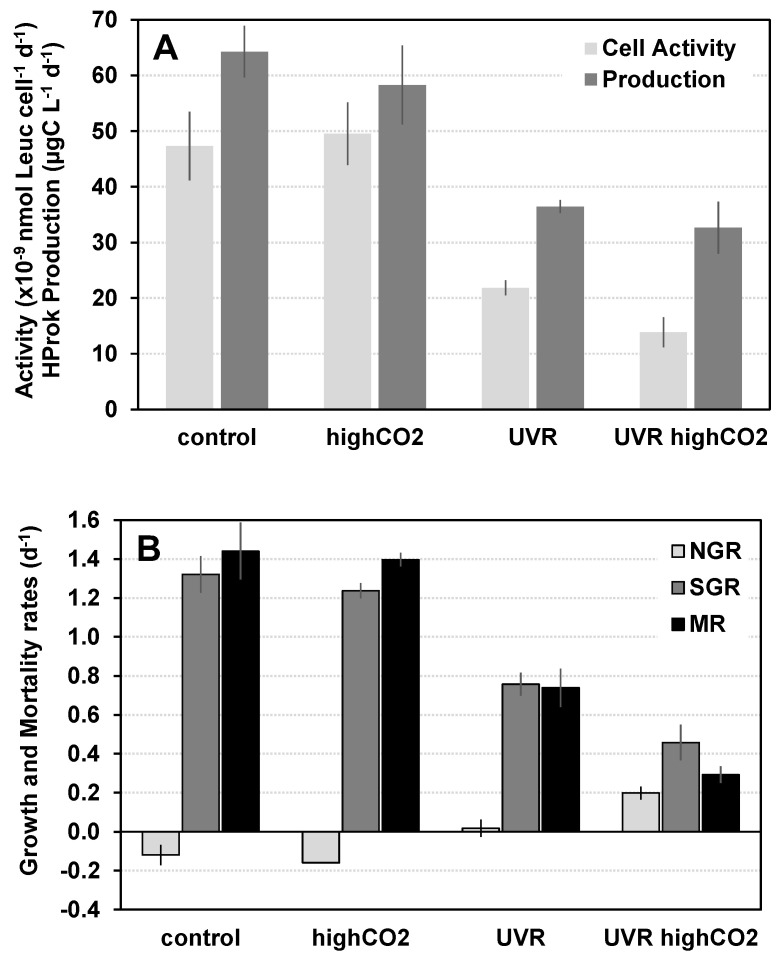
Effects of experimental manipulation of levels of CO_2_ partial pressure (CO_2_) and ultraviolet radiation (UVR), isolated and combined, on winter assemblages of planktonic heterotrophic prokaryotes (HProk) in the Ria Formosa coastal lagoon system. (**A**) cellular activity (×10^–9^ nmol leucine cell^–1^ d^–1^), and production rates (µg C L^–1^ d^–1^); and (**B**) net growth rates (NGR, d^–1^), specific instantaneous growth rates (SGR, d^–1^), and mortality rates (MR, d^–1^). Vertical lines represent ± 1 standard error.

The effects of manipulation of levels of CO_2_ and UVR (experiment 1) and CO_2_ and temperature (experiment 2) on natural assemblages of Hproks, collected in the Ria Formosa coastal lagoon, were tested in two experiments conducted during winter (January–February 2012), undertaken approximately 10 days apart. At the beginning of experiment 1, the water temperature at the sampling site was 13 °C, the chlorophyll *a* concentration was 0.9 µg L^−1^, and the Hprok abundance and biomass were 1.11 × 10^9^ cell L^−1^ and 44.3 µg C L^−1^, respectively. Under ambient conditions, average Hprok abundance, biomass, and carbon production were 8.53 × 10^8^ cell L^−1^, 21.90 µg C L^−1^, and 64.28 µg C L^−1^ d^−1^, respectively. The Hprok specific instantaneous growth (SGR) rate and mortality rate (MR) were 1.32 d^−1^ and 1.44 d^−1^, with a MR:SGR ratio of 1.09 (Table 1). The individual effects of CO_2_ enrichment were not significant for most of the Hprok variables analyzed (Figure 2, Table 2), except for mortality rate, but had a small effect size (*p* = 0.033, ω_G_^2^ = 0.05) in relation to the control. In contrast, exposure to increased UVR was associated with significant changes in all Hprok variables. Comparing with the control (ambient CO_2_ and UVR levels), individual exposure to increased UVR was associated with significantly lower Hprok production, cellular activity (leucine incorporation per cell), specific instantaneous growth rate, and mortality rates, but higher Hprok abundance and net growth rates (Figure 2, Table 1 and Table 2). Significant interactive effects of increased CO_2_ and UVR were detected only for Hprok abundance and net growth rate, with higher values for both variables under combined CO_2_ and UVR, but with low effect sizes (*p* < 0.05, ω_G_^2^ < 0.11).

**Figure 3:**
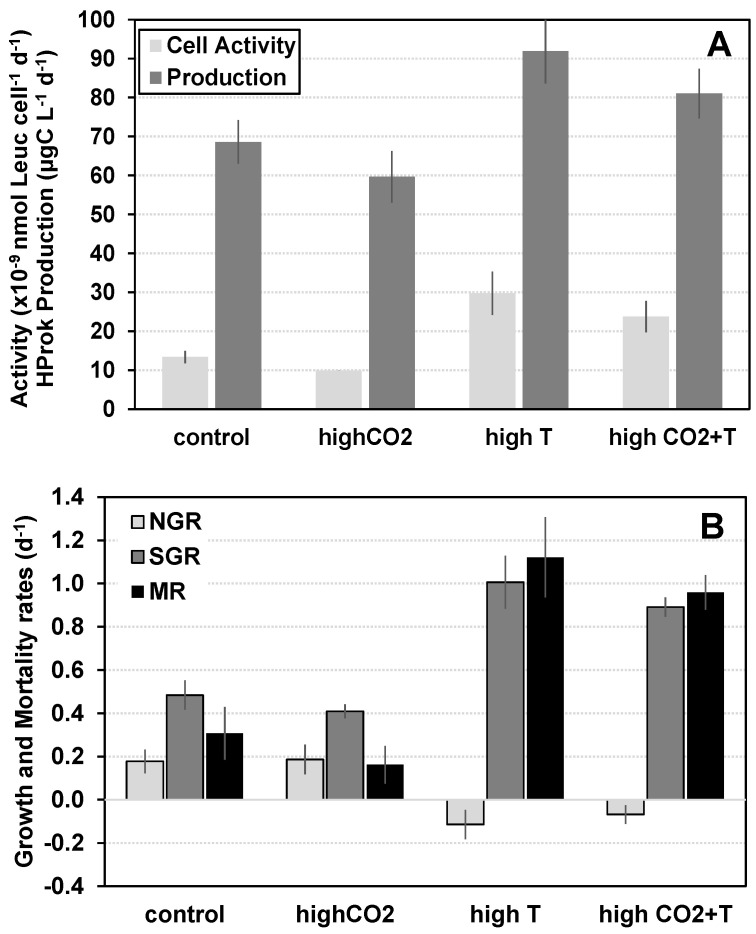
Effects of experimental manipulation of CO_2_ partial pressure (CO_2_) and water temperature (T), isolated and combined, on winter assemblages of planktonic heterotrophic prokaryotes (Hprok) in the Ria Formosa coastal lagoon system. (**A**) cellular activity (×10^−9^ nmol leucine cell^–1^ d^–1^), and carbon production rates (µg C L^–1^ d^–1^); and (**B**) net growth rates (NGR, d^–1^), specific instantaneous growth rates (SGR, d^–1^), and mortality rates (MR, d^–1^). Vertical lines represent ± 1 standard error.

At the beginning of experiment 2, water temperature at the sampling site was 10 °C, chlorophyll *a* concentration was 2.9 µg L^−1^, and Hprok abundance and biomass were 2.43 × 10^9^ cell L^−1^ and 106.1 µg C L^−1^, respectively. Under ambient conditions, average Hprok abundance, biomass, and carbon production were 3.49 × 10^9^ cell L^−1^, 116.72 µg C L^−1^, and 68.65 µg C L^−1^ d^−1^, respectively. The Hprok specific instantaneous growth (SGR) rate and mortality rate (MR) were 0.48 d^−1^ and 0.31 d^−1^, with a MR:SGR ratio of 0.27 (Table 3). The individual effects of CO_2_ enrichment were not significant for any of the Hprok variables analyzed against the control (Figure 3, Table 4). In contrast, warming was associated with significant changes in all Hprok variables. In relation to the control (ambient CO_2_ and temperature levels), individual exposure to warming (+3 °C) induced an increase in Hprok production, cellular activity, specific instantaneous growth rate, and mortality rate, but a decrease in Hprok abundance and net growth rate (Figure 3A,B, Table 3 and Table 4). No significant interactive effects of increased CO_2_ and warming were detected for any of the Hprok variables tested (Table 4).

## 4. Discussion

Our study used two short-term (2-day) microcosm experiments to evaluate the responses of natural winter HProk assemblages to abrupt perturbations in CO_2_, UVR, and temperature in the Ria Formosa coastal lagoon. Isolated increases in CO_2_ showed no effect on HProk assemblages, but when combined with increased UVR, the two variables showed a modest synergistic interactive effect on the net growth rates. Exposure to increased UVR led to significant declines in most HProk variables, whereas warming enhanced most HProk variables.

### 4.1. Ambient Conditions

Under ambient conditions, average HProk abundance, biomass, and carbon production values were similar to those typically reported for coastal systems, including the Ria Formosa [27,33,52] and other coastal lagoons [12,15,53]. The HProk specific instantaneous growth rates and mortality rates were also within the range of values reported for whole HProks in coastal lagoon systems [12,54]. A relatively high MR:SGR ratio, a proxy for global top-down control, pointed to a close coupling between HProk growth and mortality processes, usually dominated by phagotrophic protists predation and viral lysis, as previously referred for the Ria Formosa [33] and other coastal lagoon systems [12,53].

### 4.2. Effects of Increased CO_2_ on Heterotrophic Prokaryotes

The exposure of winter HProk assemblages to isolated increases in CO_2_ levels or combined with higher UVR (experiment 1) or higher temperature (experiment 2), showed no significant effects on HProk abundance, production, cellular activity, and SGR in relation to ambient CO_2_. This apparent resistance of the HProk assemblage to rapid acidification may be due to their natural acclimation to highly variable CO_2_ levels in the Ria Formosa lagoon, at both diel and seasonal scales, as usually referred for other coastal ecosystems [55,56]. The apparent lack of acidification effects on HProk abundance, production, or species composition in micro- and mesocosm manipulative experiments were reported for various coastal systems [57,58,59,60,61,62,63,64]. Yet, the lack of CO_2_ effects on HProk assemblages does not preclude the existence of species-specific responses [65,66]. Indeed, shifts in HProk species composition under elevated CO_2_ [66,67], enhanced expression of genes encoding proton pumps [68], and reductions in growth efficiency [69], in some cases without parallel changes in HProk abundance or production, have been documented.

Significant, and sometimes contrasting, effects of increased CO_2_ on marine HProk have also been reported in previous micro- and mesocosm experiments. These variable outcomes represent the integration of CO_2_ responses of HProk, phytoplankton, phagotrophic protists, and viruses, and direct and indirect effects on HProk are difficult to discriminate [11,18,19,20]. Increases in HProk production, cell-specific production, or enzyme activity under increased CO_2_ have been considered an indirect response, due to enhanced phytoplankton production and/or availability of organic matter [47,70,71,72]. During our two experiments, high CO_2_ benefited only diatoms, but showed no significant effects on phytoplankton production [30,32]; thus, this indirect stimulatory effect was probably not relevant. Direct positive acidification effects on enzyme structure and catalysis were also referred [73,74,75,76]. In contrast, negative responses of increased CO_2_ on HProk abundance, production, cell-specific production, or enzyme activity have been also reported [77,78,79].

The responses of HProk assemblages to ocean acidification are also shaped by its effects on natural mortality processes, including predation by phagotrophic protists and viral lyses [18]. In our experiments, high CO_2_ induced a moderate decline in HProk mortality only during experiment 1, and previous studies have reported variable effects. Viral abundances, replication strategies, and lyses can either be unaffected [80,81,82] or increased [19,83] due to elevated CO_2_. Bacterivory can either be unaffected [11,79,81] or reduced [79] by elevated CO_2_ levels. Indeed, reduced bacterivory can explain apparently contradictory experimental results under elevated CO_2_ (concurrent increases in HProk abundance and bulk production, but declines in cell-specific production), thus demonstrating the relevance of community-level studies [77].

HProk species composition, metabolic state [65,68,75], and other environmental determinants (e.g., nutrients: [84,85]; temperature: [11,86]; trophic regime: [81]) control the relative susceptibility/resistance of HProk assemblages and, therefore, modulate their responses to elevated CO_2_ levels. For example, mesocosm experiments undertaken in Blanes Bay revealed positive effects on HProk abundance, production, and enzyme activity during summer, under oligotrophic conditions, and no effects during winter [68,86].

### 4.3. Effects of Increased Ultraviolet Radiation on Heterotrophic Prokaryotes

In relation to the control treatment (ambient CO_2_, and PAR only), exposure to natural UVR levels (experiment 1) reduced HProk metabolism, leading to highly significant declines (ca. 50%) in production, cellular activity, and SGR. Global meta-analysis [21] and specific review studies have considered HProk amongst the most sensitive organisms to UVR stress [22]. Indeed, reductions in HProk abundance, carbon production, cell-specific activity, instantaneous growth rate, or enzyme activity after experimentally increased UVR exposure have been reported for a wide range of aquatic ecosystems, including coastal lagoons [87,88]. Despite the occurrence of rapid DNA repair processes acting during the night [89,90], the detrimental effects on HProk production, cellular activity, and SGR in the Ria Formosa may be explained by direct damages of UVR on cell deoxyribonucleic acids, proteins, and cell membranes [22]. Indirect effects linked with the photochemical alteration of labile dissolved organic matter (DOM) into recalcitrant, less bioavailable DOM, or reduced production of DOM by UVR-stressed phytoplankton [24] cannot be excluded. Indeed, during this experiment, high UVR, alone or combined with high CO_2_, showed no effects on phytoplankton production and net growth rates, but induced significant changes in assemblage composition, benefiting diatoms in relation to cyanobacteria [30]. Considering the HProk intraspecific variability in UVR susceptibility and recovery, the influence of environmental conditions [91,92,93], the commonly reported dose-dependent inhibition [91,94], and stronger direct detrimental effects of UVR on HProks in the Ria Formosa are expected during the spring–summer period.

Overall, responses of HProk natural assemblages to UVR are very complex, due to the co-occurrence of multiple direct and indirect effects on phytoplankton, HProks, and their top-down controls. Higher HProk production or activity under increased UVR has been attributed to photochemical processes improving DOM bioavailability (lability); it has even been related to an increase in the release of DOM associated with phytoplankton cell mortality induced by UVR stress [23,24,90,95]. UVR-mediated reductions in HProk mortality processes can also mitigate or even overcompensate for the influence of direct inhibitory effects of UVR on HProks. During our experiment, high UVR induced a slightly stronger decline in HProk mortality rate in relation to the instantaneous growth rate, thus leading to higher HProk net growth rates. This decline in HProk mortality can be explained by a UVR-mediated reduction in HProk bacterivory by phagotrophic protists, as previously reported in micro- and mesocosm experiments [25,96,97] or viral lyses [26]. In fact, UVR is considered a major cause of viral decay, inducing damages in virus DNA and capsid and tail proteins [98]. However, no changes in HProk viral mortality were detected under increased UVR [53], and even higher viral abundances were reported due to the induction of the lytic viral cycle in lysogenic bacteria [99]. Higher mortality also explains the minor or lack of effects of UVR on HProks [15,97,100] in tandem with a moderate natural daily UVR dose, reduced by natural mixing and resistant HProk assemblages [15].

### 4.4. Effects of Warming on Heterotrophic Prokaryotes

Contrasting with the effects of increased UVR, exposure of winter HProk assemblages to warming (ambient +3 °C), either isolated or combined with higher CO_2_ levels (experiment 2), significantly increased HProk metabolism, at both the whole assemblage (production) and individual levels (SGR, cellular activity). Similarly, higher HProk abundance, production, cell-specific activity, and growth rates under warming conditions, usually associated with direct (metabolic) effects, have been reported in micro- and mesocosm experiments addressing coastal lagoons [12,53,101]. Furthermore, the significant positive relationships between HProk abundance, production, and in situ growth rate in the Ria Formosa lagoon were previously interpreted as evidence of temperature regulation [33].

Overall, the sensitivity of HProk to temperature is variable, depending on species composition and physiological status [14,60] and bottom-up [13,102] and top-down environmental determinants. The stimulatory influence of temperature on HProk is usually higher within spatial–temporal windows of both moderate bottom-up (resource limitation) and top-down controls [103,104]. Generally, higher stimulatory effects of experimental warming on HProk have been observed during winter–early spring periods, under less oligotrophic conditions [14,104,105]. Furthermore, spatial and seasonal switches in the relative importance of temperature and substrate supply, with higher relevance of the former over coastal areas and colder periods, have also been inferred from comparative empirical approaches [106,107,108]. Considering the multiple autochthonous and allochthonous sources of organic carbon in the Ria Formosa coastal lagoon [27,33] and its mixed shallow water column, warming may probably have a more sustained stimulatory effect over the annual cycle.

During our experiment, warming induced a stronger increase in HProk mortality (ca. four-fold) in relation to instantaneous growth rate (ca. two-fold), thus leading to a decline in HProk net growth rates. HProk mortality is mostly associated with two processes: predation by phagotrophic protists and viral lyses [11,109]. In the Ria Formosa lagoon, no information is available on viral-induced mortality, and heterotrophic nanoflagellates represent the dominant bacterivores [33]. The growth rate of bacterivorous protists is usually referred as highly sensitive to warming [110], and experimental warming has been associated with increased HProk grazing in different marine ecosystems including coastal lagoons [12]. Several experimental approaches have also interpreted the absence of warming effects on HProk abundance [7,111], or even a decline of abundance or production [112], as the result of increased predation, due to the direct stimulation of bacterivores or trophic cascades [15,16]. Indirect effects of warming on virus-mediated mortality during our experiment cannot be excluded. Increases in HProk specific growth may induce a decline in the length of the lytic cycle, thus increasing viral production [113]. Conversely, higher HProk production can also promote lysogenic infection cycles with respect with lytic cycles, therefore reducing viral-induced mortality [11,114].

During our experiment, warming has also benefited the growth rate of smaller-sized phytoplankton (cyanobacteria and eukaryotic picophytoplankton), showing no effects on diatoms, but negatively affecting cryptophytes, other plastidic flagellates, and the whole phytoplankton assemblage [32]. Overall, warming apparently enhanced heterotrophic microbes and processes with respect with phytoplankton, supporting previous experiments that report shifts towards smaller [10], more heterotrophic, bacteria-based food webs under warming scenarios [11,12,13,114,115]. These differential responses of HProks and phytoplankton may be a consequence of a higher sensitivity of HProk metabolism to warming [104,116,117] and/or their improved competitive skills for nutrient uptake in relation to larger phytoplankton cells that have lower surface area to volume ratios. Integrative studies on warming effects should also consider the effects of warming on water column stratification, nutrient supply, and herbivory rates that can indirectly affect primary producers and organic carbon supply for HProk and eventually counteract the positive effects of warming [7,118].

### 4.5. Critical Analyses of the Experimental Approach

Although our experiments clearly revealed a quick and differential response of natural winter HProk assemblages of the Ria Formosa lagoon to acute climate-related perturbations, the extrapolation of results from abrupt short-term experiments to longer timescales might lead to uncertainties. Despite the advantages of manipulative microcosm-based studies, for instance, a high degree of experimental control and replication [8,9], several methodological concerns are associated with our approach. These concerns include, for example: bottle effects (possibly aggravated for low-volume microcosms); use of sudden (unnatural) changes in environmental variables and short incubations (2-day), thus minimizing and/or eliminating the influence of cell acclimation or adaptive evolution; and the exclusion of larger planktonic organisms and reduction in the number of trophic interactions within each microcosm, thus limiting the detection of potential non-direct effects (e.g., biological interactions, trophic cascades) of increases in CO_2_, UVR, and temperature on HProks [15,30,119].

The use of multi-stressor, prolonged, mesocosm-based manipulative experiments could mitigate some of these problems. Yet, since “there is no single ideal method”, future strategies aiming to mechanistically elucidate and predict climate change effects on HProks (and marine food webs) should combine multiple, complementary approaches, using realistic well-supported levels that mimic anticipated climate variability [9,85]. A comprehensive analysis of climate change impacts on HProks in the Ria Formosa coastal lagoon would also require the integration of benthic primary producers (e.g., macroalgae, saltmarshes, seagrasses, microphytobenthos), which are relevant sources of organic substrates in this ecosystem [27,33,120]. The susceptibility of HProks to climate variability is species-specific and context-dependent, considering both elevated CO_2_ [65,68,75,79,84], UVR [91,92,93,121], and warming [13,14,60,122]. Thus, future studies should also evaluate different periods over the annual cycle in the Ria Formosa lagoon. Considering the opposing effects of UVR and temperature increases detected in our study, a fully multifactorial experimental approach, specifically including the combined exposure of HProk assemblages to augmented UVR and warming, should be used.

## 5. Conclusions

Our two microcosm experiments provided quantitative information on short-term responses of winter HProk assemblages to abrupt perturbations in CO_2_, UVR, and temperature levels in the Ria Formosa coastal lagoon. These environmental changes induced differential responses. As previously hypothesized, warming significantly enhanced HProk instantaneous growth rate, carbon production, and cellular activity. In contrast, elevated UVR showed detrimental effects on these variables, and CO_2_ showed no significant effects. The effects of warming and high UVR were stronger on mortality than on instantaneous growth rates, leading to high HProk abundance and net growth rates. These responses may also represent indirect effects; for instance, the increase in HProk growth rates under UVR exposure may be caused by an increase in DOM lability or increase in DOM release due to phytoplankton mortality. These results reinforce the relevance of using natural communities to investigate climate change effects. Under future scenarios, the beneficial effects of increased warming on HProk may be partially offset by elevated UVR. A more comprehensive understanding of climate change effects on HProk assemblages should address other periods of the year and lagoon locations, apply a fully factorial approach (specifically: warming x high UVR), and use experimental strategies that allow for the analysis of other ecosystem components under prolonged periods. 

## Figures and Tables

**Table 1:** Mean values (± standard deviations or errors) for net bacterial production (µg C L^−1^ d^−1^), cellular activity (×10^−9^ nmol leucine cell^−1^ d^−1^), specific growth rate (SGR, d^−1^), net growth rate (NGR, d^−1^), mortality rate (MR, d^−1^), and chlorophyll *a* concentration (µg L^−1^) in the different experimental treatments for experiment 1.

	Net Production	Cellular Activity	SGR	NGR	MR	Chl*a*
control	64.282 ± 4.638	47.330 ± 6.171	1.321 ± 0.094	−0.120 ± 0.052	1.441 ± 0.147	2.031 ± 0.381
CO_2_	58.280 ± 7.106	49.543 ± 5.635	1.237 ± 0.094	−0.160 ± 0.004	1.396 ± 0.035	2.863 ± 0.530
UVR	36.458 ± 1.153	21.820 ± 1.342	0.757 ± 0.059	0.018 ± 0.045	0.740 ± 0.098	3.232 ± 0.522
CO_2_+UVR	32.649 ± 4.694	13.862 ± 2.681	0.458 ± 0.092	0.198 ± 0.033	0.293 ± 0.043	3.047 ± 0.744

**Table 2:** Two-way ANOVA results, with *p*-values (*p*) and generalized partial omega squared effect size values (ω_G_^2^) for the main effects of increased CO_2_ partial pressure (CO_2_), ultraviolet radiation (UVR), and interactions between these variables (CO_2_ × UVR) on the abundance, biomass, mean cell volume, carbon production rates, cellular activity (leucine uptake rates per cell), specific instantaneous growth rates (SGR), net growth rates (NGR), and mortality rates of planktonic heterotrophic prokaryotes in the Ria Formosa lagoon system during winter. Significant differences (*p* < 0.05) and large effect sizes (ω_G_^2^ > 0.70) are highlighted in bold. Ω_G_^2^ values not shown for non-significant *p*-values (>0.05).

	CO_2_		UVR		CO_2_ × UVR	
	*p*	ω_G_^2^	*p*	ω_G_^2^	*p*	ω_G_^2^
Abundance	0.092	-	**0.002**	0.56	**0.036**	0.13
Biomass	0.071	-	**0.011**	0.40	0.106	-
Net production	0.124	-	**0.003**	0.48	0.364	-
Cellular activity	0.463	-	**<0.001**	**0.86**	0.215	-
Specific growth rate	0.054	-	**<0.001**	**0.80**	0.228	-
Net growth rate	0.136	-	**<0.001**	0.63	**0.036**	0.11
Mortality rate	**0.033**	0.05	**<0.001**	**0.81**	0.064	-

**Table 3:** Mean values (± standard deviations or errors) for net bacterial production (µg C L^−1^ d^−1^), cellular activity (×10^−9^ nmol leucine cell^−1^ d^−1^), specific growth rate (SGR, d^−1^), net growth rate (NGR, d^−1^), mortality rate (MR, d^−1^), and chlorophyll *a* concentration (µg L^−1^) in the different experimental treatments for experiment 2.

	Net Production	Cellular Activity	SGR	NGR	MR	Chl*a*
control	68.650 ± 5.601	13.368 ± 1.588	0.484 ± 0.068	0.177 ± 0.055	0.307 ± 0.122	2.401 ± 0.261
CO_2_	59.624 ± 6.665	9.949 ± 0.004	0.409 ± 0.032	0.187 ± 0.069	0.162 ± 0.088	1.939 ± 0.423
T	91.987 ± 8.331	29.765 ± 5.587	1.006 ± 0.122	−0.115 ± 0.067	1.121 ± 0.185	1.829 ± 0.966
CO_2_+T	81.029 ± 6.387	23.749 ± 3.977	0.891 ± 0.044	−0.068 ± 0.043	0.959 ± 0.080	2.863 ± 0.591

**Table 4:** Two-way ANOVA results, with *p*-values (*p*) and generalized partial omega squared effect size values (ω_G_^2^) for the main effects of increased CO_2_ partial pressure (CO_2_), warming (T), and interactions between these variables (CO_2_ × T) on the abundance, biomass, mean cell volume, net production rates, cellular activity (leucine uptake rate per cell), specific instantaneous growth rates (SGR), net growth rates (NGR), and mortality rates of planktonic heterotrophic prokaryotes in the Ria Formosa lagoon system during winter. Significant differences (*p* < 0.05) and large effect sizes (ω_G_^2^ > 0.70) are highlighted in bold. ω_G_^2^ values are not shown for non-significant *p*-values (>0.05).

	CO_2_		T		CO_2_ *×* T	
	*p*	ω_G_^2^	*p*	ω_G_^2^	*p*	ω_G_^2^
Abundance	0.705	-	**0.006**	0.55	0.894	-
Biomass	0.694	-	**0.014**	0.45	0.622	-
Net Production	0.202	-	**0.016**	0.46	0.895	-
Cell activity	0.276	-	**0.007**	0.56	0.754	-
Specific growth rate	0.294	-	**<0.001**	**0.77**	0.819	-
Net growth rate	0.648	-	**0.002**	0.66	0.760	-
Mortality rate	0.299	-	**<0.001**	**0.77**	0.954	-

## Data Availability

The datasets generated during and/or analyzed during the current study are available from the corresponding author upon reasonable request.
